# A laparoscopic training model for surgical trainees

**DOI:** 10.1186/s10397-017-1028-y

**Published:** 2017-12-02

**Authors:** J. De Loose, S. Weyers

**Affiliations:** 1 General Hospital St Lucas, Groenebriel 1, 9000 Ghent, Belgium; 2 University Hospital Ghent, Ghent University, Ghent, Belgium

## Introduction

Laparoscopic training is indispensable in nearly all surgical disciplines. Nowadays, laparoscopic skills are mostly taught by simulation models [1, 2]. Training simulators allow trainees to gain experience and skills in a safe and easily accessible manner. Commercially available simulators, however, are expensive and often impractical because usage is limited in place and time. In this article, we provide directions to easily build a personal training module with a cardboard box, a tablet (ex. iPad), and laparoscopic material. Similar versions have been described in literature, but they are still too complex to build quickly [3]. Others are outdated in the sense that they need an external camera or screen to function [4].

Based on a survey among colleagues, we observed that few surgical trainees are aware of this opportunity and that they do not realize that this budget-friendly training simulator is easily accessible. These guidelines allow any surgical trainee to build his or her very own laparoscopic training model in a mere 30 min. This allows for practice at home or at the hospital during free hours and gaining more technical experience [5]. Therefore, both staff members and assistants benefit from this additional training opportunity. The possible exercises are as extensive as with professional equipment and include among others hand-eye coordination in 2D view, laparoscopic suturing, and knotting. The lack of exercising camera navigation is a shortcoming; however, this is readily learned in the OR, being the main job of the assistant as of his first year.

## Instructions

The following supplies are needed: sturdy cardboard box (measurements: approximately 50 by 50 cm and height minimum 25 cm), Stanley knife or scissors, tape, tablet computer and laparoscopic instruments.

### Instructions

**Fig. 1:**
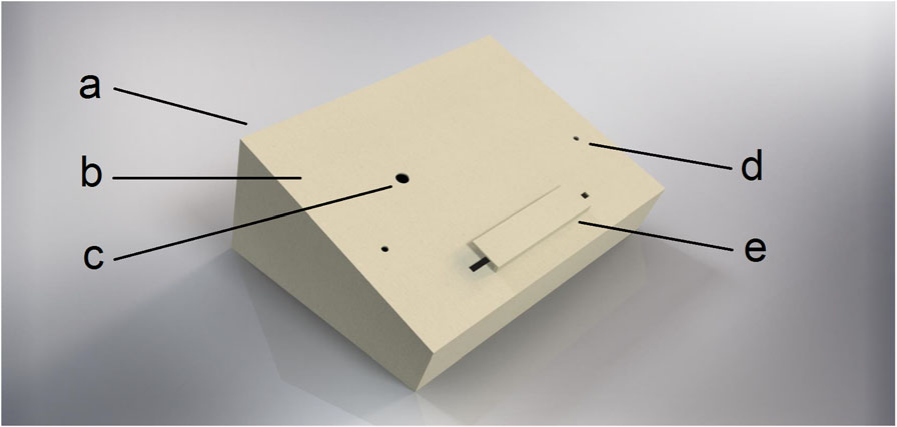
Schematic representation with indication of back plane with large opening (**a**), front plane (**b**), camera opening (**c**), trocar openings, (**d**) and iPad resting strip (**e**)

Firstly, the cardboard box is cut in a way that leaves a construction resembling a slanted roof (see Fig. 1). This is possible by making an oblique cut over one side of the box and its opposite side. The resulting large oblique plane will become the upper work space where the instruments go in and the tablet rests on. This surface should ideally make an angle of 35° to 45° with the table in order to have optimal vision when exercising.

**Fig. 2:**
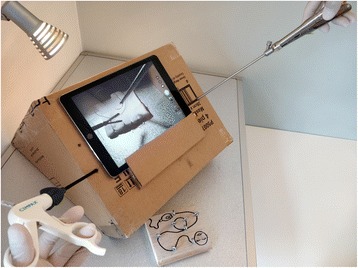
The training model as used in real-life

Secondly, a large opening is to be made in the small oblique plane at the back. This opening serves to bring in light, environmental or by a desk lamp (Fig. 2), as well as to be able to manually move training objects inside the box.

Subsequently, a thick (or double) strip of cardboard is attached horizontally in the middle of the work space area to support the tablet.

Next, an opening is cut at the place where the rear camera is situated. This varies depending on the model of the tablet and is ideally positioned centrally in the working surface, in order to obtain an as complete view of the inside of the box as possible.

Two or more narrow openings should be made at both sides next to the tablet to simulate the trocar openings.

To improve stability and durability, a piece of cardboard may be fixed underneath the box using tape.

To start practicing, the photo application of the tablet should be started, hereby projecting a continuous image of the inside of the box (Fig. 2).

Laparoscopic instruments can be bought separately, but a cheaper option is to look for secondhand or single-use instruments discarded from the operating room.

## Conclusions

This short communication provides clear step by step instructions to build a cheap laparoscopic training module. Academic and training centers could provide trainees with this module as a complementary toolbox to extend the training facilities with only a very limited budget. We are convinced that many medical students and surgical trainees will benefit from this low-threshold laparoscopic training module.
